# The right temporoparietal junction encodes efforts of others during action observation

**DOI:** 10.1038/srep30274

**Published:** 2016-07-26

**Authors:** Nobuaki Mizuguchi, Hiroki Nakata, Kazuyuki Kanosue

**Affiliations:** 1 Faculty of Sport Sciences, Waseda University, 2-579-15 Mikajima, Tokorozawa, Saitama, 359-1192, Japan

## Abstract

Smooth social interactions require a deep understanding of others’ intentions and feelings. In the present study, to investigate brain regions that respond to inference of others’ effort level, we recorded brain activity during action observation of different effort levels using functional magnetic resonance imaging (fMRI). We used a dumbbell curl movement to depict a movement requiring effort. To dissociate the factors of effort level of the actor and weight of the dumbbell, we used four combinations of dumbbell weight and actor physique: a thin actor or a built actor lifting a heavy or light dumbbell. During observation of dumbbell curls, the bilateral front-parietal action observation network (AON) was activated. This included the premotor cortices, parietal cortices, visual areas 5/superior temporal cortices (STS), amygdalae, hippocampi, right dorsolateral and ventrolateral frontal cortices. When we evaluated brain regions associated with the actor’s effort level, activity in the right temporoparietal junction (TPJ) and STS was observed. However, activity in the front-parietal AON was independent of the actor’s effort during action observation. This finding suggests that the right TPJ and STS play an important role in the inference of others’ effort levels during the observation of others’ movements.

In our daily life, many situations arise where it is important to perceive others’ intentions and feelings. The capability of understanding others’ intentions and feelings is an important aspect of smooth social interaction. In the human nervous system, two main systems have been identified as involved in the above aspects of understanding. These are the mirror neuron system (i.e. action observation network: AON) and the mentalizing system^1^,^2^,^3^,^4^,^5^,^6^. The mirror neuron system/front-parietal AON, including the premotor cortex (PM) and parietal cortices is likely to be a key substrate involved in the automatic understanding of actions performed by others^2^,^7^,^8^,^9^,^10^,^11^. For example, the front-parietal AON is associated with an inference of the “what” of an observed action (e.g. the hand grasp of a cup) as well as the “why” of an action (e.g. using the hand to grasp a cup *in order to drink*)^12^. These studies suggest that understanding the intention of others is achieved by simulation of the observed actions by utilizing the observer’s own motor system (simulation theory)^2^,^13^. On the other hand, mentalizing refers to inferring others’ mental states such as thoughts, beliefs, moral stances, and feelings. The mentalizing system has been studied using the theory of mind (ToM) task with stories and cartoons^3^,^14^,^15^,^16^. Neuroimaging studies have demonstrated that the temporoparietal junction (TPJ) plays an important role in the understanding of such mental states. During the observation of non-intended or deceptive actions, not only AON but also the TPJ is activated^17^,^18^. These findings suggest that mental states behind an other’s action affect activity in the “understanding” systems. However, it remains unclear as to whether the front-parietal AON during action observation is also related to the inference of an other’s feeling (i.e. the “how” of an action). This is due to the fact that investigations involving brain activity in association with mental states including feelings typically used cartoons or face pictures for the ToM task^7^,^19^,^20^.

An example of when we could infer the feeling of someone else, such as the effort level, would occur if we were to see an elderly adult lifting a heavy object. We would be likely to make an inference of other’s feeling of effort even when viewing their back (without watching their face). Studies utilizing transcranial magnetic stimulation (TMS) have demonstrated that corticospinal excitability during action observation of an effortful movement (lifting a heavy object) is greater than that during action observation of an effortless movement (lifting a very light object)^21^,^22^. However, these studies did not clarify whether the enhancement of corticospinal excitability reflected the effort level of the actor or the absolute weight of object. This is because the effort level and the object weight were confounding variables.

In the present study, we utilized four different movies to dissociate the effort level of the actor and the absolute weight of the object lifted, we used four types of movies: a thin actor or a built (muscular) actor lifting heavy or light weights. To investigate the brain regions that respond to the forming of an inference of other’s feelings (the actor’s effort level) with action observation, we recorded brain activity using functional magnetic resonance imaging (fMRI). We used the dumbbell curl to depict a movement requiring effort. In the case of the thin actor lifting the heavy dumbbell, the actor’s effort would be higher than for the other conditions. We hypothesized that activity in the TPJ would be related to others’ effort levels during action observation because the TPJ is the key structure for inferring other’s feelings.

## Methods

### Participants

Thirty two right-handed male participants (age: range 19–30 years, mean 23.0 ± 3.4) participated in this study. Handedness was evaluated with the Edinburgh Inventory^23^. All participants had normal or corrected normal vision. All participants received a detailed explanation of the experimental procedures before the experiment, and written informed consent was obtained from all participants. The study was approved by the Human Research Ethics committee of Waseda University. The experiment was carried out according to the principles and guidelines of the Declaration of Helsinki (1975). One participant was excluded from the analysis because he did not follow instructions while performing the task.

### fMRI data acquisition

All images were acquired using a 1.5 T MR scanner with an 8-channel head coil (Signa, General Electric, Wisc., USA). Blood oxygenation level dependent (BOLD) contrast functional images were acquired using T2*-weighted echo planar imaging (EPI) free induction decay (FID) sequences with the following parameters: TR 3000 ms, TE 50 ms, FOV 22 cm × 22 cm, flip angle 90°, slice thickness 5 mm and gap 1 mm. The orientation of the axial slices was parallel to the AC - PC line.

### Procedure

**Figure 1:**
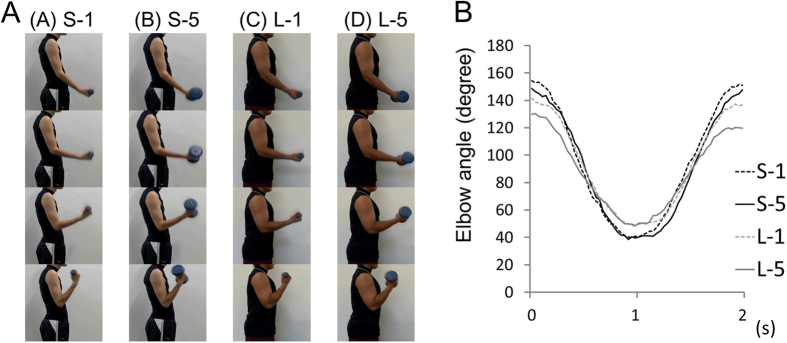
(**A**) Observed movie-clips. (**A**) a thin (slight) actor curling a 1 kg dumbbell (S-1), (**B**) a thin actor curling a 5 kg dumbbell (S-5), (**C**) a built (muscular) actor curling a 1 kg dumbbell (L-1), and (**D**) a built actor curling a 5 kg dumbbell (L-5). The pace of the dumbbell curl was 0.5 Hz. (**B**) Kinematic parameters (elbow angle) of a dumbbell burl for each condition.

The participants observed four different video-clips showing the right arm of an actor performing a dumbbell curl (Fig. 1A). These video-clips did not show the actor’s face. There were two actors and two dumbbell weights: (1) a thin (slight) actor curling a 1 kg dumbbell (S-1), (2) the thin actor curling a 5 kg dumbbell (S-5), (3) a built (muscular) actor curling a 1 kg dumbbell (L-1), and (4) the built actor curling a 5 kg dumbbell (L-5).

For the fMRI scan, a session consisted of eight alternate repetitions of the task (4 types of movement × 2 repetitions) and rest period. The order of the four dumbbell curls was randomized. The task and rest period durations were both 30 s. All participants completed six sessions. The duration of the inter-session interval was determined by each participant in order to ensure that they were neither fatigued nor sleepy. The duration of the inter-session interval was usually less than 5 min. The entire experiment always took less than 2 hours.

To present the same pace and posture for video clips for the 1 kg and 5 kg dumbbell curling, two actors were asked to maintain a smooth and constant curling movement at 0.5 Hz following the metronome sound. After a period of practice, the movement was recorded at 60 Hz using a video camera (Exilim EX-F1, Casio, Tokyo, Japan). Figure 1B shows elbow angles of one dumbbell curl for each condition. We evaluated whether the kinematics were different among the four movements using by a mixed model two-way analysis of variance (ANOVA) (within factor = dumbbell weight; between factor = actor). Then, we found a main effect of weight (F_(1,118)_ = 53.08, p < 0.01), suggesting that 5 kg dumbbell curing was slightly slower and its range of motion was slightly smaller as compared to 1 kg dumbbell curing. However, we did not find a interaction (F_(1,118)_ = 1.92, p = 0.17) nor a main effect of actor (F_(1,118)_ = 0.16, p = 0.69). Each dumbbell curl was presented repeatedly with an inconspicuous image joint in each task period (2 s × 15 times). The video clips were edited using video editing software (Adobe Premiere Pro CS5, Adobe Systems Software, CA, USA). The participants used non-magnetic goggles to observe the video-clip via a projector system (VisuaStimDigital, Resonance Technology Co, CA, USA). The participants were asked to maintain their gaze at the center of the projection and to not alter it. The participants were also asked to keep their muscles relaxed and to not think about anything throughout the entire procedure.

### Analysis

The first four volumes (12 s) of each fMRI session were discarded because of unstable magnetization. The remaining raw data were analyzed utilizing Statistical Parametric Mapping (SPM8, Wellcome Department of Cognitive Neurology, London, UK)^24^,^25^,^26^ implemented in MATLAB (Mathworks, Sherborn, MA, USA). Realigned images were normalized to the standard space of the Montreal Neurological Institute brain (MNI brain). Smoothing was executed utilizing an isotropic three-dimensional Gaussian filter with full-width at half-maximum (FWHM) of 8 mm. High-pass filters (128 s) were also applied and low frequency noise and global changes in the signals were removed.

Statistical analysis was performed on two levels. A first-level analysis was performed for each subject using a general linear model. To minimize the effects of the head motion artifacts, we included the head motion parameters estimated in the realignment procedure as regressors for each session. First, to depict the action observation network, we constructed a statistical parametric map of the t-statistic for all tasks vs. rest. Second, we depicted the regions related to each factor (actor and dumbbell weight, respectively). We analyzed the following contrasts: (S-1 vs. rest) + (S-5 vs. rest) vs. (L-1 vs. rest) + (L-5 vs. rest), (S-1 vs. rest) + (L-1 vs. rest) vs. (S-5 vs. rest) + (L-5 vs. rest). Third, we analyzed the 2 × 2 interaction: (S vs. L) × (1 kg vs. 5 kg). A conjunction analysis was also employed in order to detect brain regions of common activation for the action observation network and interacted regions.

Subject-specific contrast images of the estimated parameter were utilized to perform a second-level analysis (random-effect model)^27^. The second-level analysis was carried out in order to extend inferences of individual activation loci to those of the general population. One-sample t tests were used with a voxel-wise threshold of p < 0.001 (uncorrected) to generate cluster images. Then, we set the threshold at p < 0.05 for the cluster level after correction by the familywise error rate (FWE) for the whole brain space. The threshold for the cluster level was calculated with the Gaussian random field methods by SPM8. Small clusters were evaluated as non-significant regions. Anatomical locations and Brodmann’s areas were determined utilizing the anatomy tool box (version 1.8) of SPM. If significant activation was evaluated as ‘Not found in any probability map’, that location was excluded from description in the results section as well as in the tables.

## Results

**Figure 2:**
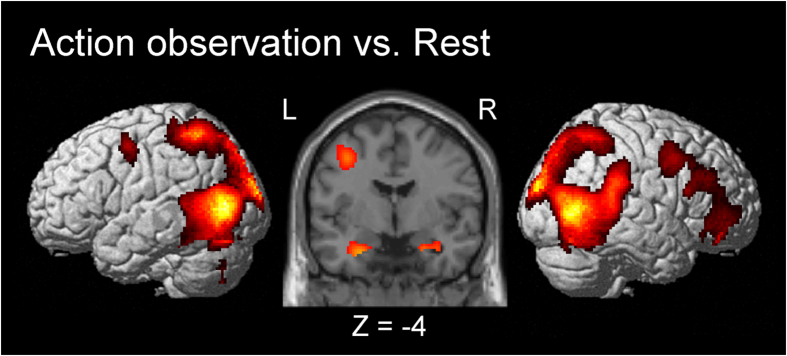
Group activation map showing activated brain regions during observation of dumbbell curls. The threshold was set at voxel level p < 0.001 (uncorrected), cluster level p < 0.05 (FWE).

We first evaluated the common neural activity associated with action observation across the conditions. During observation of the dumbbell curl, bilateral premotor cortices, temporal cortices, occipital cortices including the primary visual cortex (V1) and visual area 5 (V5), amygdalae and hippocampi were activated (Fig. 2). The right occipital cluster was extended into the right parietal cortex. In addition, the right dorsolateral prefrontal cortex (DLPFC), ventrolateral prefrontal cortex (VLPFC) and the left parietal cortex were activated (Fig. 2).

When we depicted brain regions associated with the actor (the thin actor vs. the built actor), we found two significant clusters in the left occipito-temporal area and right temporal cortex for the contrast of ((S-1 > rest) + (S-5 > rest)) − ((L-1 > rest) + (L-5 > rest)). We did not find a significant cluster for the following contrasts: ((L-1 > rest) + (L-5 >rest)) − ((S-1 > rest) + (S-5 > rest)); ((S-1 > rest) + (L-1 > rest)) − ((S-5 > rest) + (L-5 > rest)); and ((S-5 > rest) + (L-5 > rest)) − ((S-1 > rest) + (L-1 > rest)).

**Figure 3:**
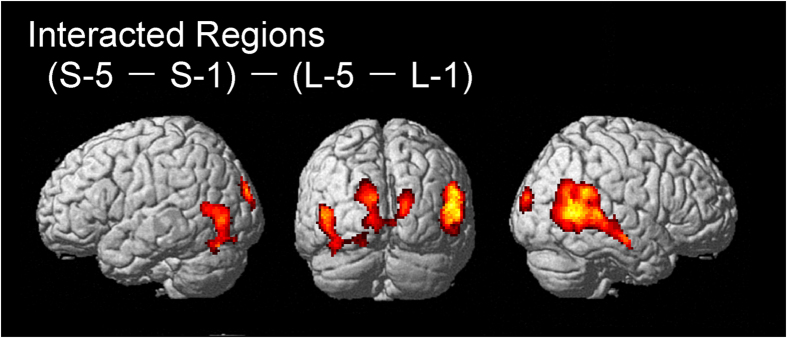
Interacted brain regions of ((S-5 > rest) − (S-1 > rest)) − ((L-5 > rest) − (L-1 > rest)). The threshold was set at voxel level p < 0.001 (uncorrected), cluster level p < 0.05 (FWE).

We found interacted brain regions: ((S-5 > rest) − (S-1 > rest)) − ((L-5 > rest) − (L-1 > rest)) in the right temporoparietal junction, bilateral temporal cortices, occipital cortices (primary visual cortex: V1) extending into the left cerebellum (Fig. 3). We did not find any interacted regions in the contrast of ((L-5 > rest) − (L-1 > rest) − ((S-5 > rest) − (S-1 > rest)).

**Figure 4:**
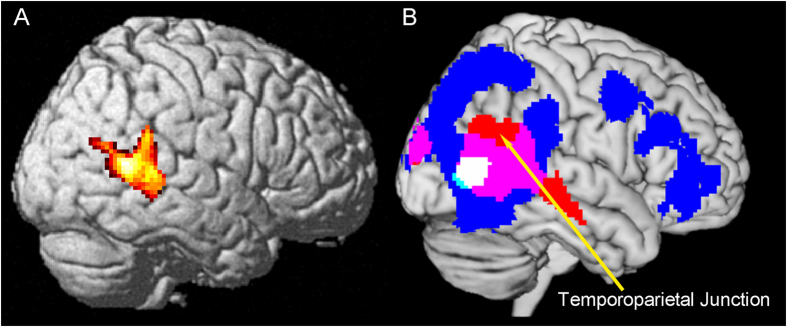
(**A**) Common regions in “action observation > rest” and “interacted regions” as identified by conjunction analysis. (**B**) Overlaid image on a ch2better template using MRIcron. Blue: action observation > rest; Red: interacted regions in “((S-5 > rest) − (S-1 > rest)) − ((L-5 > rest) − (L-1 > rest))”; Pink: “action observation > rest” and “interacted regions”; Light Blue: “action observation > rest” and “(S-1 > rest) + (S-5 > rest)) − ((L-1 > rest) + (L-5 > rest)”; White: “action observation > rest”, “interacted regions”, and “(S-1 > rest) + (S-5 > rest)) − ((L-1 > rest) + (L-5 > rest)”.

To find common regions, we conducted a conjunction analysis. We found a common region in the right posterior superior temporal sulcus (STS) (Fig. 4A). In addition, we overlaid 3 images (e.g. action observation > rest, the thin actor > the built actor, and interacted regions) (Fig. 4B).

The peaks of activity are listed in Tables 1, 2, 3, 4.

## Discussion

To investigate the brain regions responsible for inferring others’ effort levels from an observation of their body movement, we recorded brain activity during action observation of four different conditions of dumbbell curling by utilizing fMRI. First, we found that the bilateral premotor cortices, parietal cortices, V1, V5/STS, amygdalae, hippocampi, right DLPFC, and VLPFC were activated during observation of dumbbell curling (Fig. 2). Activity in the premotor and parietal cortices during action observation of dumbbell curls was consonant with that observed in the front-parietal AON/mirror neuron system^1^,^2^,^7^,^9^. In addition, activity in the occipital cortices, V5/STS, amygdalae and hippocampi during action observation was also consistent with that seen in previous studies^4^,^28^. Our main finding was that activity in the right TPJ and STS was greater during action observation of movement that involved the lifting of a heavy dumbbell by a thin actor than by a built actor. That indicates that activity in the right TPJ and STS is likely to be associated with an inference of the actor’s effort rather than with the absolute weight of the moved object.

Activity in the right TPJ was not observed in the contrast of action observation vs. rest (Fig. 4B). Therefore, activity in the right TPJ during action observation is likely specifically related to an inference of a stronger effort by someone else rather than just involving understanding of action intention. Previous studies suggest that the right TPJ can be functionally divided into anterior and posterior sections^16^,^29^,^30^. Activity in the anterior right TPJ is seen to be related to attention reorienting in the ventral attention network^31^. On the other hand, the posterior right TPJ has been described as being related to social cognition involving empathy and mentalizing^3^,^15^,^16^. Our imaging results depicted activation in both the anterior and posterior right TPJ (Figs 3 and 4B). Therefore, increased activity in this region is likely to be related to a social cognition of the actor’s effort during action observation as well as the observer’s attention to the movement.

If a weak person is lifting a heavy object, the risk of falling/injury is greater as compared with the lifting of a light object. In the former case, an observer would be likely to pay attention to the precarious movement. It has been demonstrated that voluntary attention to visual motion enhanced activity in V5^32^,^33^. Our results also showed enhancement of activity in the bilateral primary visual cortex (V1) and V5/STS during action observation involving the curling of a heavy dumbbell by a thin actor. This suggests that a greater level of attention was paid to the dumbbell curl in this case. In the present study, since we instructed the participants not to think about anything during the action observation, we assumed that the enhancement of attention to the movement would occur unintentionally. However, it is difficult to be sure whether or not the participants paid more attention to the movements intentionally or unintentionally. In addition, since the participants had to passively observe different videos in the present study, we were not able to differentiate between the effects of social cognition and attention. In the future, this question needs to be answered using an explicit task (i.e. asking the participants to explicitly judge the level of effort by actors in different videos).

The intention of imitation (i.e. social interaction) during action observation has been shown to increase activity in the front-parietal AON as compared to that which occurs during passive action observation^4^. This suggests that social interactions should enhance activity in the front-parietal AON during action observation. In the present study, however, activity in the front-parietal AON was not increased during action observation, even while a heavy dumbbell was curled by a thin actor (Fig. 3, Table 3). Therefore, attention only to the movement (without social interaction) might be insufficient to augment activity in the front-parietal AON during action observation.

A number of previous studies investigating empathy and mentalizing have utilized cartoons or face pictures^6^,^19^,^20^. We observed activity in the mentalizing system (i.e. posterior right TPJ) during action observation of effortful movements. According to previous studies, the posterior right TPJ plays an important role in the inference of others’ effort levels by observation of the movements involved in activities such as dumbbell curls. In the present study, we attempted to match the video-clips of dumbbell curls across the four conditions, but the kinematics of the observed dumbbell movements were not exactly the same (Fig. 1B). Thus, it is possible that slight differences in the movement kinematics affected activity in the right TPJ. However, as was described in the Methods section, we confirmed that no interaction with the factors of dumbbell weight and actor was observed in the kinematics. These data suggested that activity in the right TPJ cannot be explained only by the kinematics of the elbow angle. Indeed, there was no specific characteristic in the kinematics for the S-5 condition (the black line in Fig. 1B). Previous studies suggest that activity in the front-parietal AON is related to representation of action intention, kinematics, goals, and outcomes^2^,^8^,^10^. However, we did not find any significance among conditions in the front-parietal AON. These results imply that the dumbbell curl video-clips used in the present study did not differ to a great degree in kinematics, goals or outcomes. Thus, we believe that the effect of differences in movement kinematics on activity in the right TPJ was minimal.

The mentalizing system would have a hierarchy. Low-level processes such as attention reorienting are essential for appropriate higher level social cognitions^15^,^34^. Indeed, distraction of attention by increasing the cognitive load (memorizing an 8-digit number) decreases the score of self-reported empathy for subjects watching emotional pictures such as those depicting happiness or sadness^35^. We speculate that attention to the others person’s movement enhances empathy for an actor during action observation of an effortful movement. That is, activity in the both anterior and posterior right TPJ is likely necessary for an appropriate social cognition following observation of someone else’s action. Understanding the level of others’ efforts is useful for detecting the likelihood that the other person will be involved in an accident. Mishaps often occur when a task is at the limit of, or beyond, someone’s capacity. Thus, elucidating the neural mechanism that underlies the understanding of others’ efforts is important for the prevention and decrease of accidents as well as for enabling smooth social interaction.

It is well appreciated that the posterior STS is activated during observation of biological motions^36^. Activity in the right STS during action observation is modulated by social context such as indicated by emotive expressions and movements of the observed person^37^. Although it is difficult to determine how activity in the right STS was modulated, we speculate that the right anterior and/or posterior TPJ interacted with the right STS. In the present study, the right STS was consistently activated during action observation as compared to rest. Therefore, the right STS might act as a hub of the premotor-parietal AON and mentalizing system.

## Conclusions

Activity in the right anterior and posterior TPJ during observation of action with an object is likely to be associated with an actor’s effort rather than with the absolute object weight. However, activity in the premotor-parietal AON was not associated actor’s effort. This finding suggests that efforts of others during action observation are coded in the mentalizing system but not in the AON. Additionally, the right STS is likely the hub of the premotor-parietal AON and mentalizing system.

## Additional Information

**How to cite this article**: Mizuguchi, N. *et al*. The right temporoparietal junction encodes efforts of others during action observation. *Sci. Rep.* **6**, 30274; doi: 10.1038/srep30274 (2016).

## Figures and Tables

**Table 1:** Activated regions during action observation.

Region	Side	MNI coordinates			Z-score
		X	Y	Z	
Middle Orbiral Gyrus	R	42	54	−4	4.65
Inferior Frontal Gyrus	R	34	42	−20	4.64
Middle Frontal Gyrus	R	52	36	30	4.76
Precentral Gyrus	R	42	2	40	4.03
Precentral Gyrus	L	−36	−2	46	5.27
Inferior Parietal Lobule	L	−28	−46	52	5.99
Fusiform Gyrus	R	24	−70	−8	Inf
Inferior Temporal Gyrus	R	48	−52	−22	6.01
Middle Temporal Gyrus	R	52	−66	2	7.59
Middle Occipital Gyrus	L	−38	−72	4	7.59
Lingual Gyrus	R	18	−74	−4	7.47
Superior Occipital Gyrus	R	20	−88	22	7.71
Calarine Gyrus	L	0	−96	12	Inf
Superior Occipital Gyrus	L	−10	−96	18	Inf
Amygdala	R	24	−2	−26	4.69
Amygdala	L	−26	−6	−28	5.95
Hippocampus	R	24	−28	−6	6.80
Hippocampus	L	−24	−26	−10	7.07

**Table 2:** Significant regions in the ((S-1 > rest) + (S-5 > rest)) − ((L-1 > rest) + (L-5 > rest)).

Region	Side	MNI coordinates			Z-score
		X	Y	Z	
Middle Occipital Gyrus	L	−46	−80	4	4.67
Middle Temporal Gyrus	L	−48	−66	4	4.37
Superior Occipital Gyrus	L	−16	−90	34	3.71
Inferior Occipital Gyrus	L	−38	−84	−8	3.11
Middle Temporal Gyrus	R	48	−66	2	3.97

**Table 3:** Interacted brain regions of ((S-5 > rest) − (S-1 > rest)) − ((L-5 > rest) − (L-1 > rest)).

Region	Side	MNI coordinates			Z-score
		X	Y	Z	
Middle Temporal Gyrus	R	50	−40	0	4.68
Superior Temporal Gyrus	R	56	−40	22	3.73
Angular Gyrus	R	54	−64	24	3.96
Inferior Temporal Gyrus	R	48	−62	−4	3.65
Lingual Gyrus	L	−8	−74	−6	4.52
Middle Occipital Gyrus	L	−42	−76	2	3.98
Fusiform Gyrus	L	−22	−72	−16	3.58
Calcarine Gyrus	R	6	−86	0	3.34
Calcarine Gyrus	L	−6	−94	4	3.52
Cuneus	R	18	−94	12	3.85
Cuneus	L	−4	−80	16	3.25
Superior Occipital Gyrus	L	−10	−92	20	3.98
Cerebellum	L	−38	−68	−20	3.42

**Table 4:** Activated regions identified by conjunction analysis.

Region	Side	MNI coordinates			Z-score
		X	Y	Z	
Middle Temporal Gyrus	R	52	−42	2	4.62
Superior Temporal Gyrus	R	56	−42	22	3.96
